# Gonadal hormones contribute to sex differences in behavior, pathology and epigenetic modifications in the 3×Tg-AD mouse model of Alzheimer’s disease

**DOI:** 10.1186/s13293-025-00790-9

**Published:** 2025-12-01

**Authors:** Wei Song, Samantha D. Creighton, Bernadeta Michalski, Juliette Mojgani, Minesh Kapadia, Donglai Ma, Boris Sakic, Iva B. Zovkic, Margaret Fahnestock

**Affiliations:** 1 Department of Psychiatry and Behavioural Neurosciences, McMaster University, 1280 Main St. West, Hamilton, ON L8S 4K1 Canada; 2 Department of Psychological and Brain Sciences, University of Toronto Mississauga, Mississauga, ON Canada; 3 Toronto Western Hospital, Krembil Research Institute, University Health Network, Toronto, ON Canada; 4 Department of Pathology and Molecular Medicine, McMaster University, Hamilton, ON Canada

**Keywords:** Alzheimer’s disease, 3×Tg-AD mice, Gonadectomy, Sexual dimorphism, Histone variants, MacroH2A1

## Abstract

**Background:**

Sex-dependent differences in prevalence and severity are characteristics of Alzheimer’s disease (AD). Using the 3×Tg-AD mouse model, we previously reported that adult males show early behavioral dysfunction, altered epigenetic factors and lack of plaque/tangle pathology. Conversely, adult females retain more severe AD-like pathology and behavior. The present study examines whether gonadal hormones play a role in these differences in current cohorts of 3×Tg-AD mice.

**Methods:**

3×Tg-AD and wild-type mice were gonadectomized or sham-operated at 3 months of age. After behavioral phenotyping at 6 months of age, the animals were assessed for molecular markers of AD pathology and expression of genes and histone variants associated with neurodegeneration.

**Results:**

In female transgenic (AD) mice, gonadectomy resulted in poorer spatial learning performance. In contrast, in transgenic male animals, gonadectomy improved spatial learning and memory. Compared to sham-operated AD females, gonadectomized AD females exhibited enhanced expression of mouse (m) *Mapt* and *App* genes, consistent with reduced binding activity of the repressive histone variant macroH2A1 at the m*Mapt* gene, but there was no effect on Aβ_42_ or pTau181 levels. In contrast, gonadectomized AD males showed significantly increased macroH2A1 binding at the m*Psen1* promoter, reduced expression of the *App* and *MacroH2A1* genes, and reduced cortical soluble Aβ_42_ levels compared to sham-operated AD males.

**Conclusions:**

In sum, the results suggest that reduction in serum levels of female gonadal hormones impairs spatial learning capacity, whereas loss of male gonadal hormones enhances spatial learning and memory. In females, gonadectomy reduces binding of the repressive histone variant MacroH2A1 to the mouse *Mapt* gene and increases expression of the mouse *App* and *Mapt* genes without affecting Aβ_42_ or pTau181 levels. Conversely, loss of male gonadal hormones increases binding of MacroH2A1 to the mouse *Psen1* gene and decreases *App* expression and Aβ_42_ levels but has no effect on tau expression. Our work suggests that adult gonadal hormones contribute to sex differences in AD-like pathology and performance in learning and memory tasks. Moreover, sex-specific differences in AD-like pathology are partially due to the action of histone variants associated with neurodegeneration, such as macroH2A1.

## Plain language summary

**The question we studied:**

More women than men have Alzheimer’s disease, and it tends to be more severe in women - are sex hormones responsible?

**How we studied it:**

We compared what happens to male and female mice when sex hormones are removed.

**What we found:**

Removing sex hormones led to different results in male and female mice. Loss of sex hormones in males improved learning and memory and lowered amyloid-beta levels (a protein linked to Alzheimer’s disease). But in females, loss of sex hormones led to learning problems and did not affect amyloid-beta. These differences were linked to changes in how certain proteins attach to the DNA, which altered the amount of amyloid-beta differently in male versus female brains.

**Why it matters:**

These results help us understand how Alzheimer’s disease works differently in males and females. This knowledge can be the basis for developing different treatments for males and females.

## Highlights

- Loss of gonadal hormones is protective for spatial learning and memory and AD-like pathology in male 3xTg-AD (Alzheimer’s disease) model mice but is detrimental in females.
- Gonadectomy reduces binding of the repressive histone variant MacroH2A1 to the female mouse *Mapt* gene, consistent with increased expression of this gene in female, but not male, 3xTg-AD mice.
- In males only, gonadectomy increases repressive MacroH2A1 binding at the mouse *Psen1* gene, reduces expression of the mouse *App* gene, and reduces cortical soluble Aβ_42_.
- Loss of gonadal hormones may trigger sex-specific epigenetic changes, including alterations in histone variants such as MacroH2A1, that contribute via different mechanisms to sex-specific differences in pathology and behavior in Alzheimer’s disease.

## Introduction

Alzheimer’s disease (AD) is an age-related, neurodegenerative disorder that disproportionately affects women in prevalence, incidence, and severity [3, 35, 64]. One potential reason is the well-known survival advantage women have over men, but these data cannot completely explain the 2:1 ratio of women to men with AD or the increased AD severity in women [56, 69, 73, 74,104]. Although the causes of this discrepancy remain poorly understood, other factors including hormonal, genetic, and epigenetic differences between females and males have been implicated [1, 19, 22, 109]. However, the interaction of sex-specific steroids with epigenetic modifications underlying increased disease prevalence and severity in women has not been fully explored.

One reason for this gap in knowledge is the lack of an in vivo preparation suitable for testing causal relationships between sex and epigenetics in AD. Further, given that work on existing AD models rarely takes into account the 2:1 female/male bias in the incidence of AD, few have systematically assessed the importance of sex in disease progression and severity in models such as 3×Tg-AD mice [18, 50, 89]. To understand AD better, there is a need to investigate aspects of AD including sex differences and involvement of epigenetics in a valid in vivo model that mirrors the AD pathology in humans by recapitulating these aspects of the disease.

The 3×Tg-AD mouse model is an invaluable tool for studying AD pathogenesis [11] because it develops intraneuronal amyloid-beta (Aβ) by 6 months of age and neuritic plaques and neurofibrillary tangles in the cortex and hippocampus after 12 months of age [12, 83]. We showed that females exhibit more severe pathology than males [60]. Namely, the brains of 1-year-old males do not show typical AD-like neuropathology, whereas the brains of 3×Tg-AD females show Aβ plaque deposition and accumulation of hyperphosphorylated tau tangles at 1 year of age [24]. This loss of AD-like pathology in recently born male cohorts was independently confirmed by the donating investigator (https://www.jax.org/strain/004807), leading to the use of male animals being avoided in some studies [10].

Our previous work on the 3×Tg-AD mouse model provided crucial information about the existence of a relationship between sex, autoimmunity and epigenetic pathways in modulating neuropathology and behaviour in AD-like disease. We showed that in 3×Tg-AD mice, anxiety-like behavioral deficits similar to mild cognitive impairment occur from one to two months of age in both males and females, appearing as a prodrome to subsequent decline in spatial learning/memory task performance which occurs at approximately 2–3 months of age in males and 3–4 months of age in females [55, 70]. Sex-specific discrepancies in AD pathology are associated with dissimilar elevations in the expression of macroH2A1 (mH2A1), a histone variant that is upregulated during neurodegeneration [33, 47, 52]. For instance, our observation of gene expression of three histone variants in 3×Tg AD mice indicated that the expression of macroH2A1 significantly increases in 6-month-old AD males but not females compared to wild type mice, whereas in both sexes H2A.X is elevated and macroH2A2 remains unchanged [61]. Thus, an early, sex-dependent mechanism may regulate the progression and severity of disease in these mice. In this study, we aimed to determine the effect of gonadal hormones on behavior, pathology, and histone variants in adult 3×Tg-AD mice through the deprivation of gonadal hormones.

## Materials and methods

### Animals

Breeding pairs of homozygous, triple-transgenic AD mice possessing PSen1_M146V_, APP_swe_, and tau_P301L_ transgenes [83] and wild type (WT) B6.129SF2/J mice were purchased at 6 weeks of age from the Jackson Laboratory (Bar Harbor, ME). All mice were bred and maintained in our vivarium, where they were group-housed (for breeding pairs, 2–3 mice/cage; for cohorts, 4 mice/cage) and kept under standard laboratory conditions and 12-hour reverse light cycle: light phase 7 P.M.−7 A.M., room temperature ∼22 °C, humidity ∼62%, low fat rodent chow and tap water available *ad libitum*, and Beta Chip bedding changed twice per week. All animal-related protocols were performed in accordance with the rules and regulations of the Canadian Council of Animal Care and approved by the local Animal Research Ethics Board.

Transgene constructs: Human *APP* (695 isoform) cDNA harboring the Swedish double mutation (KM670/671NL) and human *MAPT* carrying a mutation (P301L) were expressed under control of the mouse Thy1.2 expression cassette, resulting in overexpression of APP and tau [83]. For *Psen1*, the human PS1 M146V knock-in mutation of the mouse *Psen1* gene results in expression of a presenilin-1 protein with the human familial Alzheimer’s disease-linked mutation PS1M146V at normal physiological levels (https://www.jax.org/strain/004193; [46]).

### Surgery

At 3 months of age, the mice were gonadectomised under isoflurane gas anesthesia to remove either bilateral testicles or ovaries to deplete endogenous gonadal hormones. Non-gonadectomised controls underwent sham surgeries. All mice were individually housed post-surgery until endpoint. Body weight was measured biweekly post-surgery as an important monitoring parameter.

### Behavioral testing

After acclimatization and 5 days of habituation to experimenters, mice underwent a battery of behavioral tests pre- and post-surgery at 2.5 and 6 months of age, respectively. The following measures and paradigms were used sequentially: basic neurological reflexes, basket test, Rotarod test (these were used to screen for non-memory-related neurological deficits), step-down test, and Morris water maze (MWM). The last two tests were used only for post-operative animals, considering their potential impact on memory training.

Reflexes: To assess basic neurological function, mice were tested for clasping reflex, visual placing reflex, and geotaxis. All mice with surgeries, including all genotypes and sexes, showed good reflexes comparable to mice without surgeries.

Basket test: The test assesses muscle strength, motor coordination and sensorimotor deficits. A mouse was placed in the center of a short basket box (8” high, made of wire mesh) which was then slowly inverted. The time it took for the mouse to fall was recorded by stopwatch.

Rotarod test: This test uses a rotating bar (Med Associates, Fairfax, Vermont), to examine muscle strength and sensorimotor coordination. Three 5-min trials daily were given over three days, with latency and speed at fall recorded. The duration (seconds) on the rod was automatically recorded via computer.

Step-down test: The step-down test is designed to assess the readiness of a mouse to escape from an elevated platform by descending onto a firm, dark surface (usually black cardboard) in a brightly-lit room [5, 99]. This task assesses acrophobia and, as such, it is proposed to evoke an anxiety-like response to height and/or bright light [70]. A small platform (10 × 8 cm) made of wire mesh was placed on top of a 7 cm-high rectangular Pyrex glass bowl. The mouse was placed on the wire mesh, and the latency to step down with all four paws was recorded during a 10-min trial.

Morris Water Maze (MWM): The MWM is commonly used to assess visual-spatial learning and memory in rodents [27]. An 8-day MWM protocol was used to habituate the animals to the apparatus, assess their sensorimotor capacity, and examine their ability to form a spatial map [59]. The polyethylene swimming pool (dia. = 105 cm, H = ~ 60 cm, San Diego Instruments, San Diego, CA), filled with tap water at ∼24 °C, had a circular platform made of clear Plexiglas (dia. 15 cm) positioned in the northwest quadrant. Mice were trained in four cue trials (Day 1), with the platform above the water surface and a blue cylinder on the top. Starting positions (north, south, east, or west quadrant) were randomly chosen on each day. The cue trial was considered completed when the mouse found the platform or if a 2 min trial expired, in which case the mouse was placed on the platform. In both cases the mouse remained on the platform for 45–50 s to form a spatial map. On Days 2–5, the platform was hidden, and blocks of acquisition trials (4 trials/day) were given over four days. To examine whether a spatial learning strategy was employed in solving the task, a 2-min probe trial was given on Day 6, and time spent in the northwest quadrant was measured. The cued reversal trial consisted of placing the platform in the quadrant opposite to its original location (southeast quadrant), and then two acquisition trials were performed with the platform visible and two with the platform submerged. The following measures were recorded for cued and acquisition trials: latency to find the platform (either visible or submerged), distance traversed, and swimming speed.

### Genotyping

The protocol was adapted from The Jackson Laboratory (Bar Harbor, Maine) to detect the inserted, mutated human tau gene. Crude extraction of DNA from 2 to 3 mm mouse tail was performed with REDExtract-N-Amp™ Tissue PCR Kit (Sigma-Aldrich, Oakville, ON, Canada) according to manufacturer’s instructions. Amplifications were performed in a total volume of 15 µl containing 7.5 µl of REDExtract-N-Amp PCR mix (Sigma), 0.5 µM of each primer, and 3 µl of mouse tail extract as template. The primer sequences were from The Jackson Laboratory and ordered from IDT (Coralville, IA, USA). The expected polymerase chain reaction (PCR) product of the transgene (fragment of P301L) was 254 *bp* long, and the internal positive control (IPC), a fragment of the mouse interleukin 2 gene, was 324 *bp* long. Primer information is listed in Table 1. The PCR protocol consisted of 10 cycles of touchdown (1 °C drop every 2 cycles/min of the annealing temperature from 65 °C to 60 °C [44], followed by 28 cycles of 30 s at 94 °C, 1 min at 60 °C, and 1 min at 72 °C. The final extension cycle was 10 min at 72 °C. The size of the amplification products was determined by agarose gel electrophoresis.

**Table 1:** Primer sequences used for PCR and qRT-PCR

Gene	Accession	Forward primer	Reverse primer	Note	Product size
*Mapt* (M)	XM_001198824.1	5’-GTC CTC GCC TTC TGT CGA TT-3’	5’-GCT GTG GGG GAG ACT CTT TT-3’	For cDNA (gene expression)	153 bp
*Mapt* (M)	NC_000077.7	5’-GTC CTC GCC TTC TGT CGA TT-3’	5’-GAG GGG TGG CAG GAG GA-3’	For gDNA (mH2A1 binding)	91 bp
*App* (M)	NM_001198824.1	5’-GGA CCA CTC GAC CAG GTT CTG-3’	5’-GAT GAG GCG CCT TTG TTC G-3’	For cDNA (gene expression)	154 bp
*App* (M)	NC_000082.7	5’-AGA GAC AGA CAG ACT CTT GGG T-3’	5’-TCC TTA GGT GAT GCT CTG GAT G-3’	For gDNA (mH2A1 binding)	106 bp
*Mapt* (Tg)	NM_001377265.1	5’-GCA ATT CCT TTT GAT TCT TTT T-3’	5’-GGT TGA CAT CGT CTG CCT G-3’	For cDNA (gene expression)	205 bp
*App* (Tg)	NM_201414.3	5’-GCG CTG GAG GTA CCC A-3’	5’-TGC AGG TTT TGG TCC CTG AT-3’	For cDNA (gene expression)	139 bp
*Thy1.2* (M)	NC_000075.7	5’-CAG CAC TGG GCT CTT GAG TT-3’	5’-CCG TTC CGT CTC CAG ACA AG-3’	For gDNA (mH2A1 binding)	192 bp
*Psen1* (M)	NM_001362271.1	5’-TAT ACC CGG AAG GAC GGT CA-3’	5’-CAG GCG TGG ATG ACC TTG TA-3’	For cDNA (gene expression)	179 bp
*Psen1* *(M)*	NC_000078.7	5’-TGC TAG GAA TCC CAG TCC GA-3’	5’-AAG GAG CAC ACA CGA GGA AC-3’	For gDNA (mH2A1 binding)	136 bp
*MacroH2A1* (M)	NM_001159513.1	5’-CCC GGA AGT CTA AGA AGC AGG G-3’	5’-AGG ATT GAT TAT GGC CTC CAC C-3’	For cDNA (gene expression)	188 bp
*β-actin* (M)	NM_007393.5	5’- AGA TCA AGA TCA TTG CTC CTC CT-3’	5’- ACG CAG CTC AGT AAC AGT CC-3’	For cDNA (gene expression)	174 bp
*Gapdh* (M)	NM_001289726.1	5’-GTG GAG TCA TAC TGG AAC ATG TAG-3’	5’-AAT GGT GAA GGT CGG TGT G-3’	For cDNA (gene expression)	150 bp
*Mapt*^*3*^ (Tg)	NM_005910.6	5’-TGA ACC AGG ATG GCT GAG-3’	5’-TTG TCA TCG CTT CCA GTC C-3’	JAX code_ 15,238^1^ & 15,239^2^	255 bp
*IPC* (M)	AH_001969.2	5’-CTA GGC CAC AGA ATT GAA AGA TCT-3’	5’-GTA GGT GGA AAT TCT AGC ATC ATC C-3’	JAX code_ oIMR7338^1^ & oIMR7339^2^	325 bp

^1^forward primer, ^2^reverse primer; ^3^*Mapt *primers for genotyping, M: mouse, Tg: human transgene, IPC: internal positive control

### Sample collection

Mice were anesthetized with an intraperitoneal injection of a ketamine/xylazine mixture at 7 months of age. Blood (∼1 ml) was collected from the chest cavity within 15 s of severing the vena cava into a 1.5 ml Eppendorf tube. The blood was left to clot on ice for 20 min, and then serum was isolated by centrifugation at 12,298 ×g for 5 min at room temperature. Blood vessels were intracardially perfused with ∼120 ml of phosphate buffered saline (PBS) over 5 min. Extracted brains were wet weighed, dissected, and frozen until use.

### Serum hormone levels

The levels of testosterone and estradiol were measured in undiluted serum samples. For males, the Calbiotech Mouse/Rat Testosterone ELISA (Calbiotech, El Cajon, CA) was used according to the manufacturer’s instructions, with the standard curve extended to include testosterone levels from 0.05 to 12 ng/ml. Estradiol levels in serum of females was assayed using the Double Antibody RIA 17β-Estradiol kit (MP Biomedicals, Irvine, CA USA). The manufacturer’s protocol was followed with 2 changes: (1) volume of all reagents and samples were halved because of limited serum volume, (2) 5 pg/ml was added to the estradiol standard curve as the lowest detectable concentration.

### Amyloid-beta and Tau proteins

Extraction of soluble tau and Aβ species was based on published methods [72, 98]. In brief, frozen cortical samples (approx. 100 mg) were sonicated in ∼1 ml (1:10 volume) Tris-buffered saline (TBS) pH 7.4 with protease inhibitors (Halt™ Protease Inhibitor Cocktail, Thermo Fisher Scientific, Waltham, MA), 1 mM 4-(2-aminoethyl) benzenesulfonyl fluoride hydrochloride (AEBSF, Bio Basic, New York, NY), and phosphatase inhibitors (PhosSTOP EASYpack™ phosphatase inhibitor tablets, Roche, Mississauga, ON), and kept on ice for 5–10 min. For soluble Aβ analysis, TBS homogenates were centrifuged for 20 min at 14,000 ×g at 4 °C, and supernatants (S1) were collected, aliquoted, and frozen at − 80 °C until use. For analysis of soluble tau, TBS homogenates were centrifuged for 20 min at 27,000 ×g at 4 °C, and supernatants (S1) were collected and frozen for subsequent use. Protein concentrations in each fraction were measured using a detergent-compatible protein assay (Bio-Rad Laboratories, Mississauga, ON, Canada). Transgenic (human) Aβ42 protein levels in TBS-soluble S1 fractions were measured using the Amyloid beta 42 Human ELISA Kit (Invitrogen, San Diego, CA) per manufacturer’s instructions. Transgenic (human) total tau and phospho-tau 181 were assessed with the Human Total Tau ELISA kit and the Human Tau (Phospho) [pTau181] ELISA Kit (Invitrogen, San Diego, CA). Concentrations were acquired with a MultiskanGO and SkanIt software (Thermo Scientific, Nepean, ON, Canada) at 450 nm. These ELISA kits recognize only transgenic human amyloid-beta and tau, and mouse (endogenous) gene products were not detected. Values are presented as pg of the human gene product in 1 µg or 1 mg of total protein, depending on transgene production.

### macroH2A1 chromatin Immunoprecipitation (ChIP)

ChIP and PCR were performed as previously described [79]. Briefly, cortical samples were incubated in 1% formaldehyde for 10 min at 37 °C, and 1.25 M glycine was added to quench the reaction. Next, samples were washed with PBS, and SDS lysis buffer was added to all samples prior to sonication at 40% power, 6× for 10 s with 50 s rest using a Bioruptor^®^ Plus sonication device (Diagenode, Denville, NJ) with a model CL-18 probe (Fisher Scientific, Ottawa, ON, Canada). Samples were centrifuged at 17,000 ×g for 10 min, aliquoted, and diluted with ChIP dilution buffer (MilliporeSigma, Oakville, ON, Canada). Samples were treated with 20 µl of Millipore Protein G magnetic beads and 1 µl of anti-macroH2A1 (Cell Signalling Technology, Danvers, MA, USA) or anti-histone H3 (MilliporeSigma) antibody overnight at 4 °C. The next day, samples were washed sequentially with low-salt (20 mM Tris-HCl pH 8.0, 2 mM EDTA pH 8.0, 0.1% SDS, 1% Triton X-100, 150 mM NaCl) and high-salt (20 mM Tris-HCl pH 8.0, 2 mM EDTA pH 8.0, 0.1% SDS, 1% Triton X-100, 500 mM NaCl) buffers, LiCl (10 mM Tris-HCl pH 8.0, 1 mM EDTA, 0.25 M LiCl, 1% NP-40, 1% Sodium Deoxycholate), and then Tris-EDTA (TE) buffer (10 mM Tris-HCl, 1 mM EDTA, pH 8.0) and incubated with rotation for 5 min between washes. Immune complexes were extracted from both ChIP and input samples using proteinase K (0.625 mg/ml in TE buffer) and heated at 65 °C for 2 h followed by 95 °C for 10 min before purification with a PCR Purification Kit (Bio Basic Inc., Markham, ON, Canada). Primers (Table 1) were designed to detect specific sequences in the promoter regions of **(1)** mouse *Arc*,* Fos*,* Mapt*,* App*, and **(2)** mouse *Thy1.2* expression cassette for human *App*_*swe*_ *P301L* [83], and **(3)** mouse *Psen1* promoter and *Psen1*_M146V_. ChIP data were calculated as % input, then normalized against the (histone H3) control.

### RNA expression

RNA was extracted from mouse cortical samples in TRIzol using the EZ-10 spin column total RNA extraction kit (BioBasic, Markham, ON, Canada). Complementary DNA was synthesized using a high-capacity cDNA Reverse Transcription Kit (Applied BioSystems, Waltham, MA, USA). Primers are shown in Table 1. Data were normalized to the geometric mean of β-actin and GAPDH [60, 79]. Data were then normalized to the same sex WT Sham, defined as “relative expression”.

### Statistical analysis

Raw data were analyzed using SPSS 20 software package (SPSS Inc., Chicago, IL) and Prism-GraphPad (GraphPad Software, San Diego, CA). Analysis of variance (ANOVA) was used in the overall analysis, with Genotype (3×Tg-AD vs. WT), Sex (male vs. female) and Treatment (gonadectomy vs. sham) as between-group factors, and Trial or Age as within-group factors, where applicable. To reveal modulatory effects of gonadectomy on behavior, 3×Tg-AD pathology, and epigenetic factors (histone variants), the focus of this statistical analysis was on the significant interaction between Genotype and Treatment (represented as Genotype × Treatment in the following text), or interaction between Genotype and Sex and Treatment (represented as Genotype × Sex × Treatment in the following text). Given our previous extensive behavioral, neuropathological and immunological characterization of this mouse model [60, 61, 70], other significant main effects were less important for the current research question. The significance level was set at *p* ≤ 0.05. For statistically significant ANOVAs, *post hoc* tests were carried out, corrected by controlling the false discovery rate (FDR) (two-stage step-up method of Benjamini, Krieger and Yekutieli). Graphs indicate mean values ± SEM, with significant differences of q ≤ 0.05, q < 0.01, q < 0.001, and q < 0.0001 shown as *, **, *** and ****, respectively.

## Results

### Body weight

**Fig. 1:**
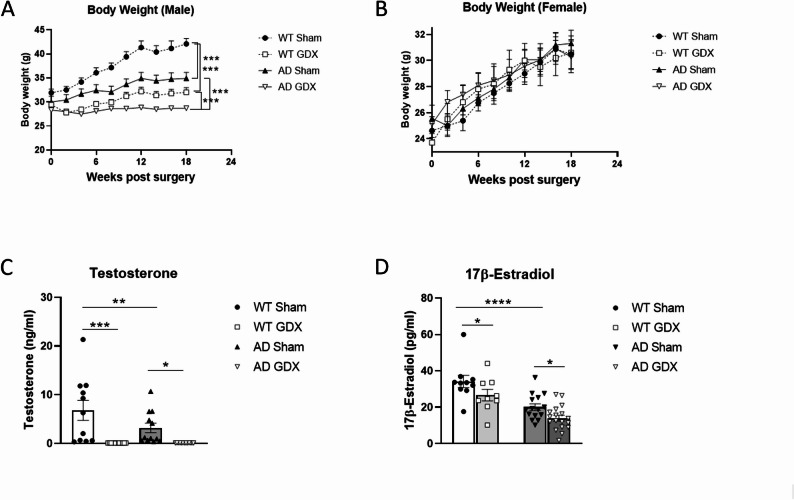
Body weight and hormone levels of male and female mice following GDX or sham surgeries. **(A)** Body weight for each male mouse was recorded on the day of surgery, at 3 weeks post-surgery, and continuing every 2 weeks thereafter until the endpoint. GDX mice gained less weight than sham-operated mice, and WT mice were heavier than AD mice. Group size: WT male Sham = 12, WT male GDX = 14; AD male Sham = 12, AD male GDX = 14. **(B)** Body weight for each female mouse was recorded with the same time course as male animals. All animals gained weight at the same rate following surgery. Group size: WT female Sham = 8, WT female GDX = 10; AD female Sham = 16, AD female GDX = 21. **(C)** Serum levels of testosterone in males at endpoint, measured by ELISA. GDX eliminated serum testosterone. WT male Sham = 11; WT male GDX = 12; AD male Sham = 12; AD male GDX = 13. **(D)** Serum 17β-estradiol in females at endpoint, measured by radioimmunoassay. GDX significantly reduced serum estradiol. WT female Sham = 10, WT female GDX = 9; AD female Sham = 15; AD female GDX = 18. *q < 0.05, ***q < 0.001. Error bars represent SEM

Body mass is an important parameter to estimate malnutrition and post-surgery complications, especially infection [65]. As shown in Fig. 1A and B, mice either gained weight or did not lose weight over time [Time: F(9, 670) = 118.7, *p* < 0.0001], indicating no post-operative complications, malnutrition or infection. Gonadectomised (GDX) male mice were lighter than sham-treated mice, and male AD mice were lighter than male WT mice [Fig. 1A, Time × Treatment: F(9, 423) = 19.70, *p* < 0.0001; Time × Genotype: F(9, 423) = 12.25, *p* < 0.0001, q < 0.0001 between WT Sham and GDX or AD Sham and GDX groups], indicating that this genetic model displayed growth retardation and that removal of male gonadal hormones indiscriminately affected both the 3×Tg-AD and WT control animals. In females, no significant differences were found between any groups (Fig. 1B). GDX did not cause any changes in brain weight in either males or females (data not shown).

### Serum testosterone and estradiol

Gonadectomy (GDX) eliminated serum testosterone in male WT and AD mice compared to the animals with sham surgeries [Fig. 1C, Treatment: F(1, 21) = 21.42, *p* = 0.0001, Genotype: F(1, 23) = 2.95, *p* = 0.099, Genotype × Treatment: F(1, 21) = 2.998, *p* = 0.098, q = 0.0003 between male WT Sham and GDX groups, q = 0.026 between male AD Sham and GDX groups]. GDX also significantly reduced serum estradiol in female WT and AD animals compared to those with sham surgeries (Fig. 1D, Treatment: F(1, 20) = 11.42, *p* = 0.003, Genotype: F(1, 28) = 28.87, *p* < 0.0001,       Genotype × Treatment: F(1, 20) = 0.12, *p* = 0.735, *post hoc:* q = 0.03 between female WT Sham and GDX groups, q = 0.03 between female AD Sham and GDX groups). Levels of serum testosterone in Sham male AD mice and serum estradiol in Sham female AD mice were lower than in Sham WT animals (Fig. 1C, D and q = 0.02 for testosterone between AD Sham and WT Sham males, q < 0.0001 for estradiol between AD Sham and WT Sham females).

### Behavioral performance

#### Basket test

**Fig. 2:**
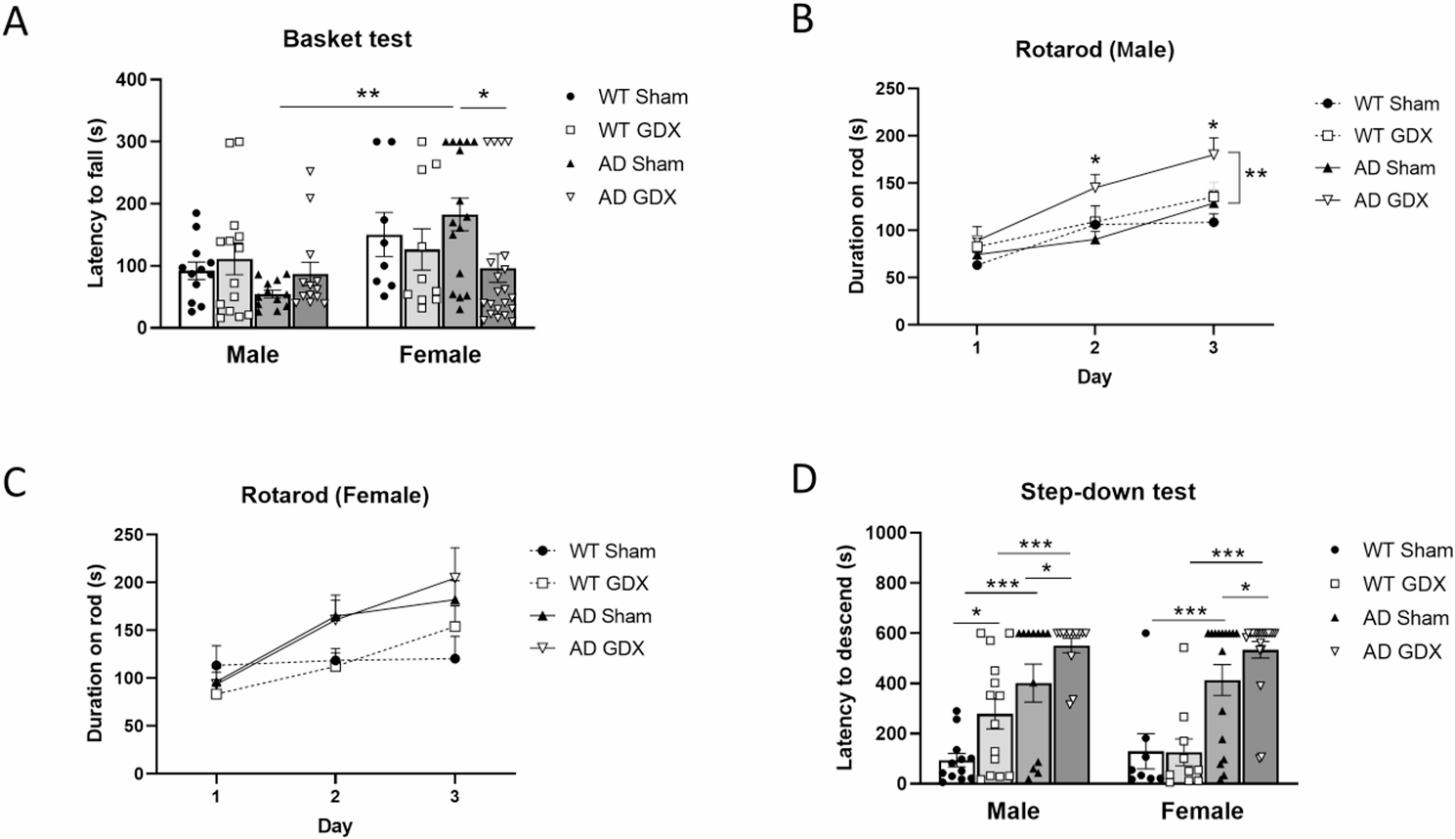
*Tests of muscle strength*, *motor coordination*, *and anxiety in male and female mice following GDX or sham surgeries*. **(A)** Basket test for muscle strength. Female AD mice performed better than male AD animals in the Basket test, although this difference was abolished by GDX. Performance measured as latency (in seconds) before a mouse dropped to the bottom of the basket. Group size: WT male Sham = 12, WT male GDX = 14; AD male Sham = 12, AD male GDX = 13; WT female Sham = 8, WT female GDX = 10; AD female Sham = 16, AD female GDX = 21. **(B)** Rotarod test for motor coordination and balance post-surgery. GDX male AD mice performed better on the Rotarod test than sham-treated male AD mice. The rotarod test was performed for each male mouse on 3 consecutive days, and the duration (seconds) on the rod was automatically recorded by computer. Animals showed a training effect over the consecutive 3 days of trials. **(C)** GDX had no effect on female AD mouse performance on the Rotarod test. The rotarod test was performed for each female mouse on 3 consecutive days, and the duration (seconds) on the rod was automatically recorded by computer. Animals showed a training effect over the consecutive 3 days of trials. Group sizes: WT sham = 8, WT GDX = 10; AD sham = 16, AD GDX = 21. **(D)** Step-down test to assesses acrophobia, an indicator of anxiety. In the Step-down test, AD mice exhibited a longer step-down latency, suggesting anxiety, than WT animals, and this anxiety was generally increased by GDX. Group sizes the same as in **A**. *q < 0.05, **q < 0.01, ***q < 0.001. Error bars represent SEM

For the basket test, the mice displayed a sex difference dependent upon treatment [Fig. 2A, Sex: F(1, 59) = 6.35, *p* = 0.01, Genotype: F(1, 39) = 0.44, *p* = 0.51, Treatment: F(1, 59) = 0.77, *p* = 0.38, Sex × Treatment: F(1, 59) = 4.97, *p* = 0.03]. Specifically, following sham surgery, female AD mice performed better than male AD animals, whereas WT animals did not display any sex differences **(**Fig. 2A, q = 0.007 between AD male and AD female groups, q = 0.39 between WT male and female groups). Following GDX in AD females, this sex difference was abolished (Fig. 2A, q = 0.81 between male AD GDX and female AD GDX groups), indicating that removal of female gonadal hormones reduces muscle strength only in AD females (Fig. 2A, q = 0.04 between AD female Sham and GDX groups).

#### Rotarod test

GDX male AD mice performed better on the Rotarod than sham-treated male AD mice [Fig. 2B, Trial: F(2, 141) = 18.04, *p* < 0.0001, Genotype: F(2, 141) = 4.25, *p* = 0.04, Treatment: F(1, 141) = 11.68, *p* < 0.001, q = 0.008 between AD Sham and GDX]. In contrast, GDX had no effect on female AD mice [Fig. 2C, Trial: F(2, 153) = 8.35, *p* < 0.001, Genotype: F(1, 153) = 5.82, *p* = 0.02, Treatment: F(1, 153) = 0.026, *p* = 0.87. q = 0.95 between AD Sham and GDX].

#### Step-down test

AD mice exhibited a longer step-down latency, suggesting “acrophobia”, than WT animals, regardless of sex [Fig. 2D, Sex: F(1, 98) = 0.64, *p* = 0.43, Genotype: F(1, 98) = 68.85, *p* < 0.0001, Treatment: F(1, 98) = 8.59, *p* = 0.004]. GDX generally prolonged step-down latency, suggesting increased anxiety (q = 0.01 between male WT Sham and GDX groups, *p* = 0.04 between male AD Sham and GDX groups, q = 0.47 between female WT and GDX, q = 0.04 between female AD Sham and GDX).

#### Morris water maze

**Fig. 3:**
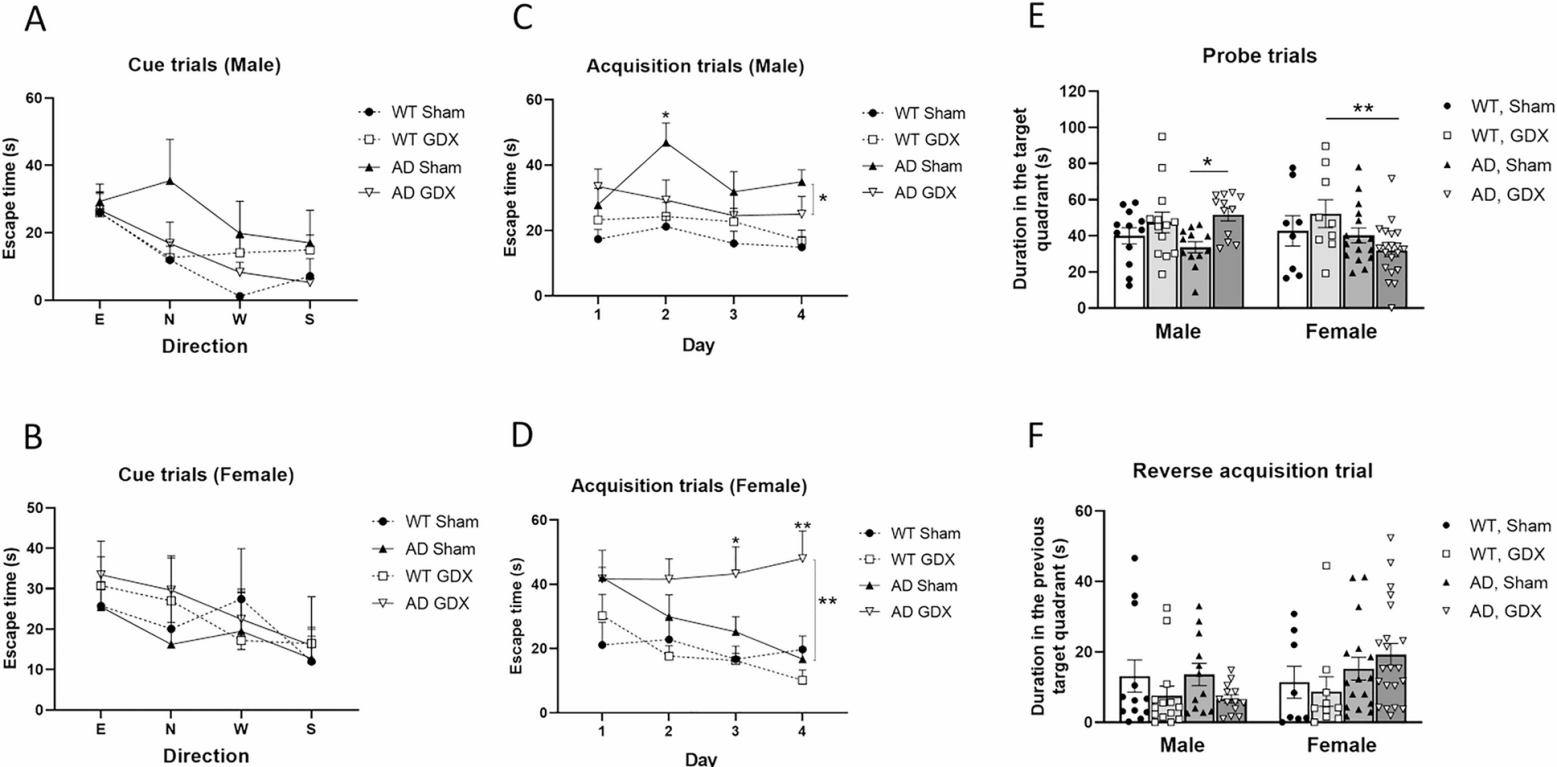

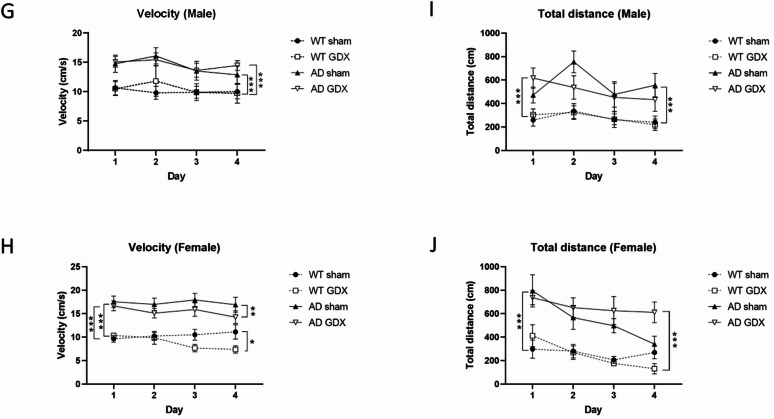
Morris Water Maze test for spatial learning and memory in male and female mice following GDX or sham surgeries. **(A)** Cue trial to check males’ swimming ability and vision. All male groups showed comparable performance in cued trials, suggesting AD mice have no apparent deficits in visual acuity or motor capacity, and GDX did not impact these parameters. **(B)** Cue trial to check females’ swimming ability and vision. All female groups showed comparable performance in cued trials, suggesting AD mice have no apparent deficits in visual acuity or motor capacity, and GDX did not impact these parameters. **(C)** Acquisition trial to assess spatial learning in male mice. In acquisition trials, male Sham AD mice displayed longer escape latencies than the other groups, indicating they did not show signs of spatial learning. **(D)** Acquisition trial to assess spatial learning in female mice. In acquisition trials, female GDX AD mice displayed longer escape latencies than the other groups, indicating they did not show signs of spatial learning. **(E)** Probe trial to assess spatial memory. Probe trials demonstrated that GDX increased performance of male AD mice only. **(F)** Reversal trial to assess cognitive flexibility. In reversal trials, all mice spent similar amounts of time in the previous target quadrant, implying that neither sex nor removal of gonadal hormones affects cognitive flexibility. **(G)** Male swimming velocity in the acquisition trial. WT mice exhibited significantly lower velocities than AD mice, and this difference was unaffected by removal of gonadal hormones. **(H)** Female swimming velocity in the acquisition trial. WT mice exhibited significantly lower velocities than AD mice, and removal of gonadal hormones significantly decreased the velocity of both WT and AD mice. **(I)** Total swimming distance of males in the acquisition trial. WT mice exhibited significantly shorter swim distances than AD mice, and this difference was unaffected by removal of gonadal hormones. **(J)** Total swimming distance of females in the acquisition trial. WT mice exhibited significantly shorter swim distances than AD mice. This difference was unaffected by removal of gonadal hormones in WT animals, whereas AD mice with GDX surgery exhibited significantly longer swim distances than sham-operated animals. Group sizes: WT male Sham = 12; WT male GDX = 14; AD male Sham = 12; AD male GDX = 13. WT female Sham = 8; WT female GDX = 10; AD female Sham = 16; AD female GDX = 21. *q < 0.05, **q < 0.01, ***q < 0.001. Error bars represent SEM

Cue trials examined visual acuity and swimming ability. As depicted in Fig. 3A and B, all mice could see well and could learn to mount a visible platform, i.e. latency to find the platform for escape become shorter with increasing number of trials [Trial: F(3, 216) = 10.65, *p* < 0.0001]. No difference was observed between trials and genotype, sex, or treatment [Trial × Genotype: F(3, 175) = 0.33, *p* = 0.81, Trial × Sex: F(3, 216) = 0.38, *p* = 0.77, Trial × Treatment: F(3, 216) = 0.066, *p* = 0.98].

Acquisition trials assessed spatial learning capacity via allocentric navigation. AD and WT mice showed different spatial learning capacities following GDX or sham surgery [Day × Genotype × Sex × Treatment: F(3, 156) = 3.23, *p* = 0.023; Genotype: F(1, 156) = 19.56, *p* < 0.0001, Genotype × Sex × Treatment: F(1, 156) = 4.52, *p* = 0.04]. A more specific group comparison revealed that although GDX had no effect on WT mice, neither sham-treated AD males nor GDX AD females showed signs of spatial learning (Fig. 3C, D and q = 0.04 between AD Sham and AD GDX in male and q = 0.003 in female). GDX worsened AD females’ and enhanced AD males’ performance.

Probe trials evaluated if the mice employed a spatial search strategy. There was a significant interaction of Genotype × Sex × Treatment [Fig. 3E, Sex × Genotype × Treatment: F(1, 37) = 4.36, *p* = 0.04], indicating that GDX improved spatial memory in AD males but not AD females [Genotype × Treatment for male: Treatment: F(1, 45) = 8.41, *p* = 0.006, q = 0.006 between AD sham and AD GDX; Genotype × Treatment for female: Treatment: F(1, 49) = 0.01, q = 0.92].

Reversal trials tested the cognitive flexibility of the mice by relocating the platform to the opposite quadrant. A three-way ANOVA (Genotype × Sex × Treatment) indicated no significant main effects [Genotype: F(1, 59) = 0.85, *p* = 0.36; Sex: F(1, 59) = 1.44, *p* = 0.24; Treatment: F(1, 39) = 2.52, *p* = 0.12]. Removal of gonads did not change cognitive flexibility in either male or female AD mice (Fig. 3F).

Velocity and total distance in acquisition trials may help to assess spatial learning capacity. In male animals, wild type mice had lower swim velocity than AD mice [Fig. 3G, Genotype: F(1, 92) = 82.82, *p* < 0.0001. q < 0.0001 between WT and AD (GDX or sham)], but shorter distance traveled before escape [Fig. 3I, Genotype: F(1, 92) = 61.73, *p* < 0.0001. q < 0.0001 between WT and AD (GDX or sham)] than AD mice, indicating that the difference in spatial learning capacity between WT and AD mice reflected by the escape time (Fig. 3C) was independent of swim velocity. These differences were not changed by removal of gonadal hormones. In female animals, similar to males, wild type mice also had lower swim velocity [Fig. 3H, Genotype: F(1, 204) = 91.53, *p* < 0.0001. q < 0.0001 between WT and AD (sham)] and shorter distance traveled [Fig. 3J, Genotype: F(1, 204) = 48.04, *p* < 0.0001. q < 0.0001 between WT and AD (sham)] than AD mice. In contrast, the velocity decreased in both WT and AD female animals with removal of gonadal hormones [Fig. 3H, Treatment: F(1, 204) = 5.735, *p* = 0.018. q = 0.01 between WT sham and GDX, q = 0.006 between AD sham and GDX], possibly contributing in small part to the dramatically increased escape time in Fig. 3D. There was a trend towards an increase in distance in female AD mice following GDX, but the difference was not statistically significant (Fig. 3J, q = 0.054 between AD sham and AD GDX).

### Neuropathology

#### Soluble amyloid beta

**Fig. 4:**
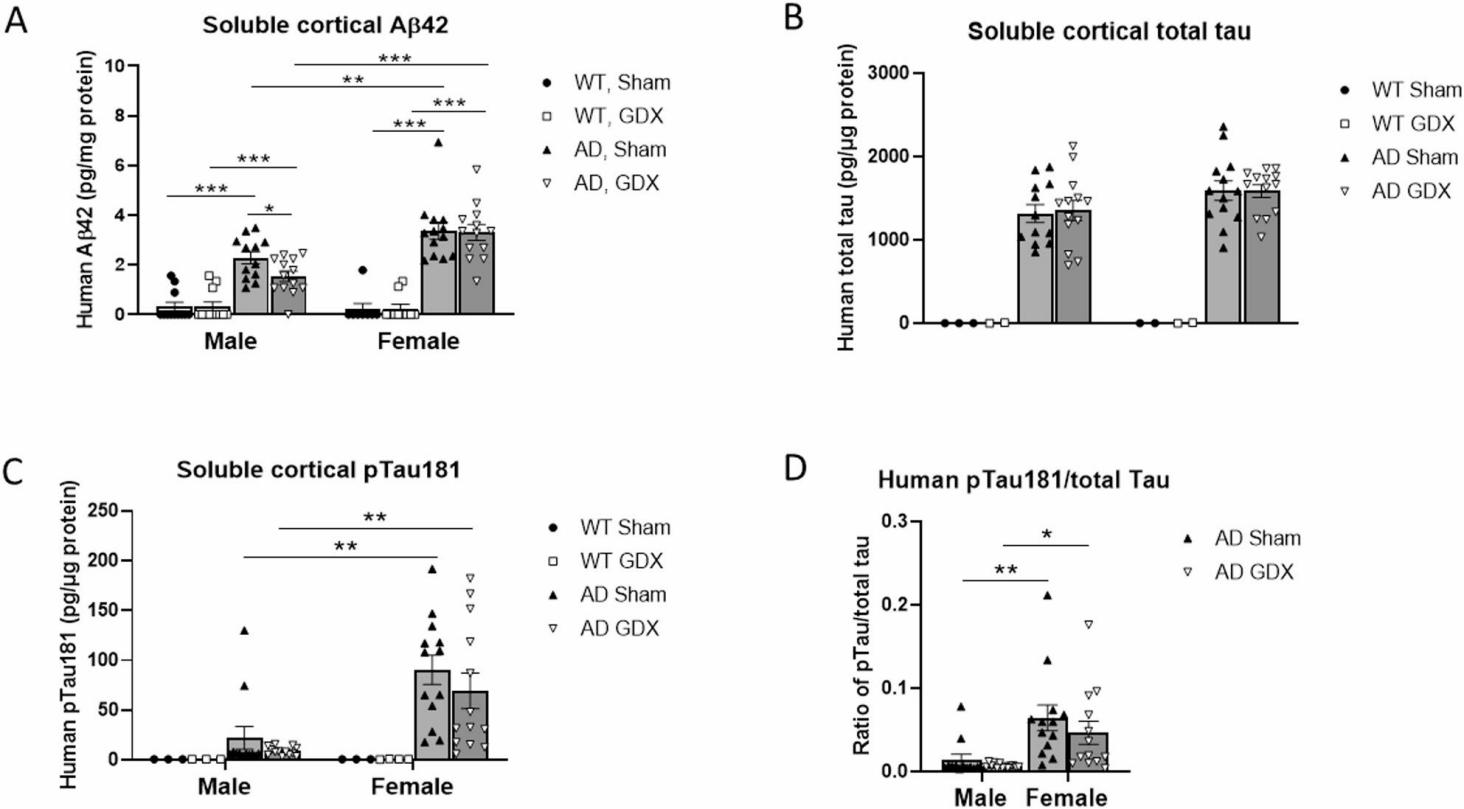
Cortical amyloid-β and phosphorylated tau pathology in male and female 3xTg-AD mice post GDX or sham surgeries. **(A)** Cortical TBS-soluble Aβ_42_ in AD and WT mice. Female AD mice had greater soluble Aβ_42_ levels than male AD mice, and GDX reduced soluble Aβ_42_ in male AD mice only. Group sizes: male WT Sham = 12; male WT GDX = 12; male AD Sham = 12; male AD GDX = 13; female WT Sham = 8; female WT GDX = 10; female AD Sham = 12; female AD GDX = 13. **(B)** Female AD mice had greater cortical TBS-soluble total human tau levels than male AD mice, and GDX had no effect on tau levels. **(C)** Female AD mice had greater cortical TBS-soluble human phospho-tau 181 (pTau181) levels than male AD mice, and GDX had no effect on pTau181 levels. Group sizes for **B** and **C**: male WT Sham = 3; male WT GDX = 2–3; male AD Sham = 12; male AD GDX = 13; female WT Sham = 2–3; female WT GDX = 2–4; female AD Sham = 13, female AD GDX = 13. **(D)** Female AD mice had a greater ratio of pTau181 to total tau than male AD mice, and GDX did not affect the ratio. Group sizes: male AD Sham = 12; male AD GDX = 13; female AD Sham = 13, female AD GDX = 13. *q < 0.05, **q < 0.01, ***q < 0.001. Error bars represent SEM

Studies indicate that soluble β-amyloid (sAβ) oligomers, rather than their fibrillary aggregates, contribute to AD pathogenesis [68]. In addition, Aβ_42_ is more toxic than Aβ_40_ [49]. As shown in Fig. 4A, AD mice displayed elevated sAβ_42_ levels in TBS-soluble fractions of the cortex compared to WT [Genotype: F(1, 38) = 189.9, *p* < 0.0001]. This increase in soluble human Aβ_42_ was greater in AD females than males [Sex: F(1, 47) = 12.88, *p* < 0.001; Genotype × Sex: F(1, 38) = 19.82, *p* < 0.0001]. A 2-way ANOVA for male mice only revealed that there was a strong trend towards significance for a Treatment main effect and a Genotype x Treatment effect. *Post hoc* analysis showed that GDX male AD mice exhibited significantly less sAβ_42_ than sham-treated male AD mice [Genotype: F(1, 45) = 62.9, *p* < 0.0001, Treatment: F(1, 45) = 3.25, *p* = 0.078; Genotype × Treatment: F(1, 45) = 3.53, *p* = 0.067. q = 0.012 between male AD sham and GDX].

#### Soluble total Tau and phospho-Tau 181

Both human total tau and pTau181 antibodies were specific for human tau, as in WT samples, tau was below the detection limit [Fig. 4B, Genotype: F(1, 5) = 129.5, *p* < 0.0001]. GDX did not affect total tau levels in either sex [Fig. 4B, Treatment: F(1, 47) = 0.06, *p* = 0.81; Sex × Treatment: F(1, 5) = 0.02, *p* = 0.93]. Compared to total tau expression, pTau181 content was much lower in AD males than in AD females [Fig. 4C, Sex: F(1, 56) = 5.91, *p* = 0.02]. This was also true for the ratio of pTau181/total tau [Fig. 4D, Sex: F(1, 47) = 16.97, *p* = 0.0002, q = 0.005 between male AD sham and female AD sham groups, q = 0.01 between male AD GDX and female AD GDX groups]. However, similar to total tau, GDX did not affect pTau181 content in either sex [Treatment: F(1, 47) = 1.33, *p* = 0.26; Sex × Treatment: F(1, 47) = 0.22, *p* = 0.64].

### Epigenetic changes

#### MacroH2A1 gene expression

**Fig. 5:**
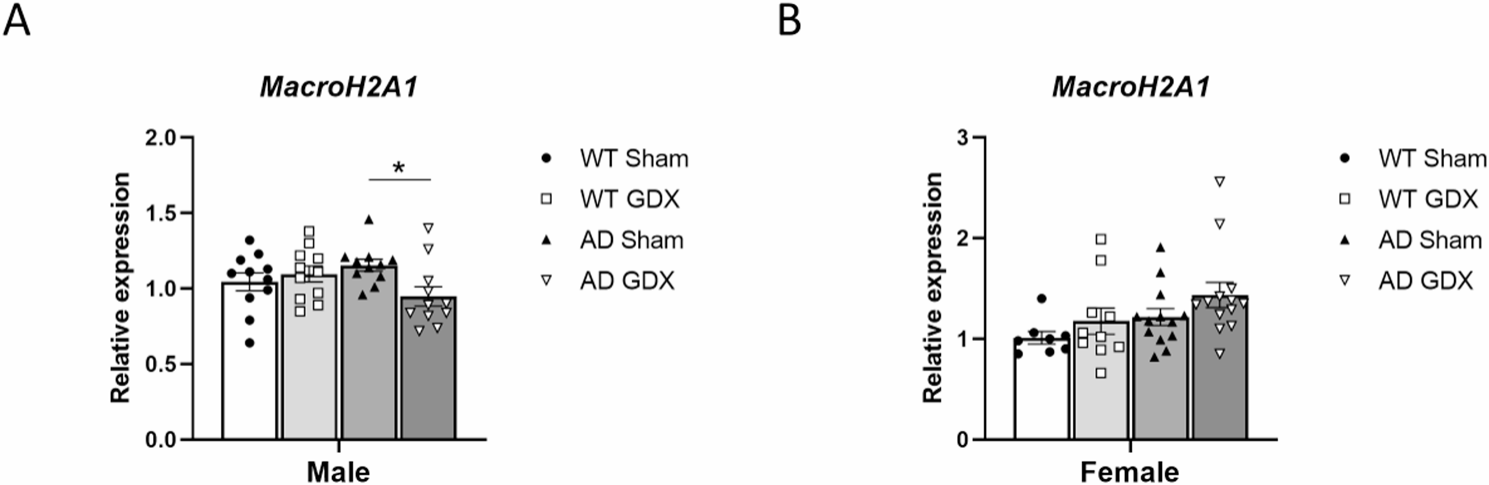
Expression of *macroH2A1* mRNA by qRT-PCR in 3xTg-AD mouse cortex following GDX or sham surgeries. Data were normalized to the geometric mean of β-actin and GAPDH. Data were then normalized to the same sex WT Sham, defined as “relative expression”. **(A)** GDX significantly decreased cortical *macroH2A1* expression in AD males. Group sizes: males: *n* = 11 in all groups. **(B)** There was a strong trend towards an increase in GDX AD females compared to Sham. Group sizes: females: WT Sham = 8, WT GDX = 10, AD Sham = 13, AD GDX = 13. *q < 0.05 between male AD GDX and male AD Sham groups; q = 0.055 between female AD GDX and female AD Sham groups. Error bars represent SEM

The *macroH2A1* gene (formerly *H2afy*) encodes the histone variant macroH2A1, which is upregulated in Huntington’s disease and Alzheimer’s disease [47, 52]. The level of *macroH2A1* mRNA expression in male mouse cortex was subject to a Genotype × Treatment interaction [F(1, 20) = 4.69, *p* = 0.04], whereby GDX reduced *macroH2A1* levels in AD but not WT male mice [Fig. 5, q = 0.02 for AD Sham vs. GDX), q = 0.29 for WT Sham vs. GDX]. In contrast, *macroH2A1* expression in females did not differ significantly between sham-operated AD and WT animals [Fig. 5, Genotype: F(1, 21) = 2.19, *p* = 0.15]. However, there was a strong trend towards elevated *macroH2A1* expression in female AD GDX animals compared to AD Sham females [Fig. 5, Treatment: F(1, 19) = 5.29, *p* = 0.03, q = 0.055 between female AD Sham and female AD GDX]. There were no significant genotype-associated differences in *macroH2A1* mRNA expression in males or females.

#### MacroH2A1 binding at mouse genes

**Fig. 6:**
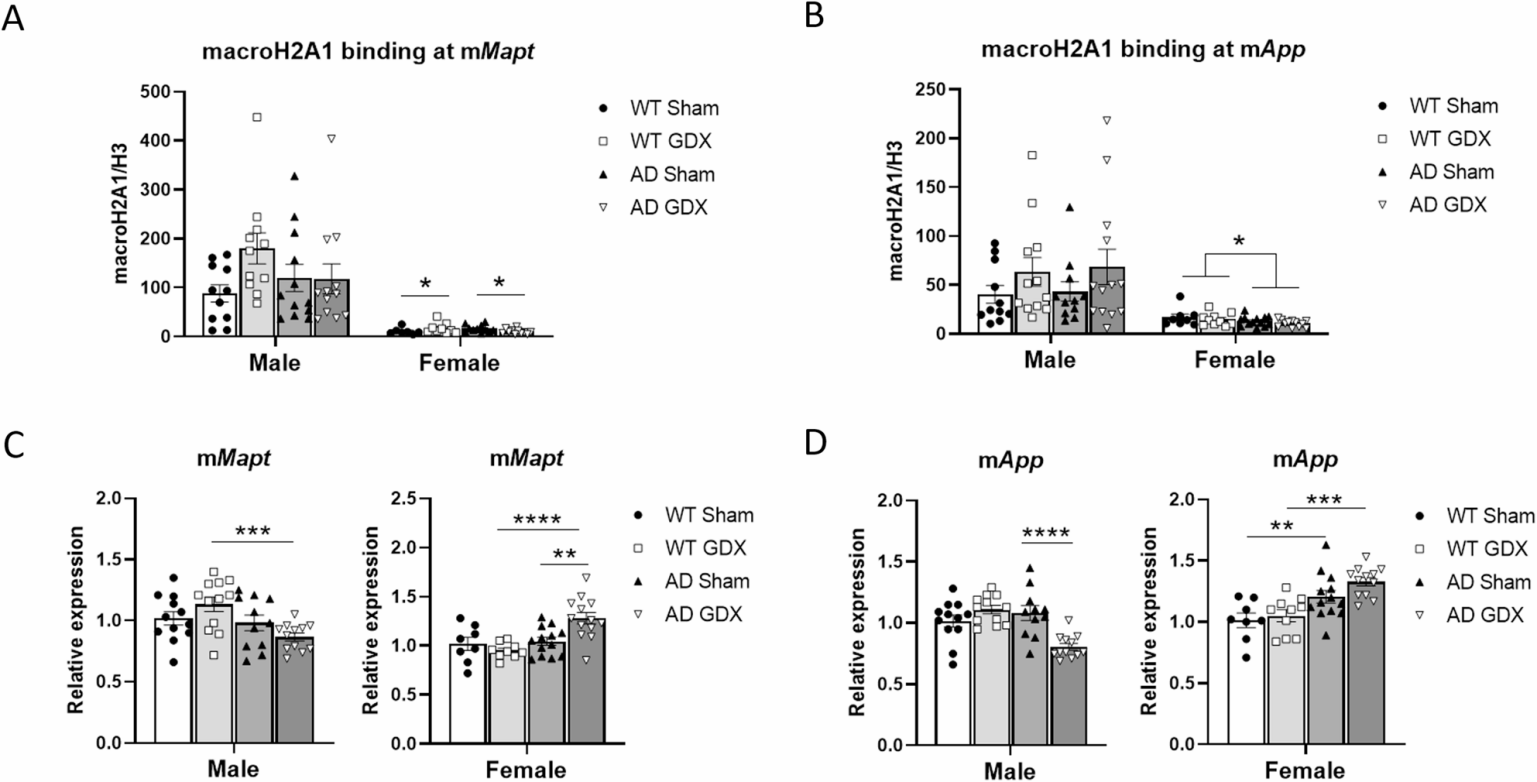
Histone variant macroH2A1 binding at mouse genes and targeted mouse gene expression in male and female 3×Tg-AD mice following GDX or sham surgeries. **(A)** Binding of macroH2A1 at the gene body of m*Mapt* by macroH2A1 ChIP-qPCR demonstrated that GDX reduced binding in female AD mice only. Group sizes: WT male Sham = 11, WT male GDX = 11; AD male Sham = 12, AD male GDX = 12. WT female Sham = 7, WT female GDX = 9; AD female Sham = 11, AD female GDX = 11. **(B)** Binding of macroH2A1 at the gene body of m*App* by macroH2A1 ChIP-qPCR demonstrated that GDX had no effect on binding. Group sizes: WT male Sham = 11, WT male GDX = 12; AD male Sham = 11, AD male GDX = 13. WT female Sham = 8, WT female GDX = 10, AD female Sham = 13, AD female GDX = 12. **(C)** m*Mapt* gene expression in mouse cortex by qRT-PCR demonstrated increased expression in male WT mice compared to male AD mice (left), but increased expression in female AD mice compared to female WT mice (right). GDX increased m*Mapt* gene expression in female AD mice only (right). Group sizes: WT male Sham = 12, WT male GDX = 12; AD male Sham = 11, AD male GDX = 12. WT female Sham = 8, WT female GDX = 9, AD female Sham = 13, AD female GDX = 13. Data were normalized to the geometric mean of β-actin and GAPDH. Data were then normalized to the same sex WT Sham, defined as “relative expression”. **(D)** m*App* gene expression in mouse cortex by qRT-PCR demonstrated that GDX decreased m*App* expression in AD males (left) but did not significantly change it in AD females (right). Group sizes: WT male Sham = 12, WT male GDX = 12; AD male Sham = 11, AD male GDX = 12. WT female Sham = 8, WT female GDX = 10, AD female Sham = 13, AD female GDX = 12. Data were normalized to the geometric mean of β-actin and GAPDH. Data were then normalized to the same sex WT Sham, defined as “relative expression”. *q < 0.05, **q < 0.01, ***q < 0.001, ****q < 0.0001. Error bars represent SEM

MacroH2A1 binding at promoter regions of (mouse) m*Mapt* and m*App* was significantly higher in males than females [Fig. 6A and B, Sex: F(1, 76) = 53.43, *p* < 0.0001 for m*Mapt*; F(1, 45) = 24.42, *p* < 0.0001 for m*App*]. There were no genotype- or treatment-associated differences in macroH2A1 binding in males [m*Mapt*: Genotype, F(1, 76) = 0.31, *p* = 0.61; Treatment, F(1, 76) = 2.14, *p* = 0.14. m*App*: Genotype, F(1, 37) = 0.002, *p* = 0.969; Treatment, F(1, 45) = 1.74, *p* = 0.19]. There was a significant Genotype × Treatment interaction for macroH2A1 binding at m*Mapt* for females [Fig. 6A, F(1, 16) = 9.71, *p* = 0.007], with GDX resulting in reduced macroH2A1 binding in AD females and increased macroH2A1 binding in WT females (Fig. 6A, q = 0.049 between AD Sham and AD GDX; q = 0.049 between WT Sham and WT GDX). In contrast, macroH2A1 binding on m*App* was reduced in AD females compared to WT females irrespective of GDX [Fig. 6B, Genotype: F(1, 39) = 4.59, *p* = 0.04, Treatment: F(1, 39) = 1.56, *p* = 0.22, Genotype × Treatment: F(1, 39) = 0.001, *p* = 0.97].

#### Targeted mouse gene expression

To determine if genes exhibiting differential macroH2A1 binding are also differentially expressed, we conducted qRT-PCR in cortical tissue for the same genes that were analyzed for macroH2A1 binding. The presence of macroH2A1 in reconstituted nucleosomes at promoter regions was previously shown to inhibit gene expression [15].

ANOVAs for each sex revealed there was a Genotype × Treatment interaction for m*Mapt* expression in males [F(1, 43) = 4.61, *p* = 0.04] and in females [F(1, 39) = 8.40, *p* = 0.006]. Upon removal of gonadal hormones, m*Mapt* expression decreased in AD males and increased in AD females compared with GDX WT animals, but there was no such genotype difference in sham-operated animals (Fig, 6 C, for males: q = 0.001 between WT and AD GDX, q = 0.34 between WT and AD Sham; for females: q < 0.0001 between WT and AD GDX, q = 0.38 between WT and AD Sham). Interestingly, neither AD nor WT males exhibited any significant changes in m*Mapt* expression following GDX compared with sham-treated mice (Fig. 6C, q = 0.15 between sham-treated and GDX males for both WT and AD mice). In contrast, in females, GDX increased m*Mapt* expression in AD mice but not in WT mice (Fig, 6 C, q = 0.001 between AD sham-treated and AD GDX, q = 0.41 between WT sham-treated and WT GDX), consistent with macroH2A1 binding status.

Male AD mice exhibited lower m*App* expression following GDX than male sham-treated AD mice (Fig. 6D, Genotype: F(1, 43) = 7.48, *p* = 0.009; Treatment: F(1, 43) = 4.28, *p* = 0.045, Genotype × Treatment: F(1, 43) = 18.01, *p* = 0.0001. q = 0.0001 between GDX and sham-treated AD males). In contrast, GDX had no significant effect in female AD mice. m*App* expression was increased in sham-treated AD females compared to sham-treated WT mice and in GDX-treated AD mice compared to GDX-treated WT mice, but there was no difference between GDX and sham-treated females (Fig. 6D, Genotype: F(1, 16) = 27.01, *p* < 0.0001, Treatment: F(1, 23) = 2.18, *p* = 0.15, Genotype × Treatment: F(1, 16) = 0.96, *p* = 0.34. q = 0.01 between WT and AD sham-treated females; q = 0.0007 between WT and AD GDX-treated females; q = 0.13 between GDX and sham-treated AD females).

#### MacroH2A1 binding on transgenes

**Fig. 7:**
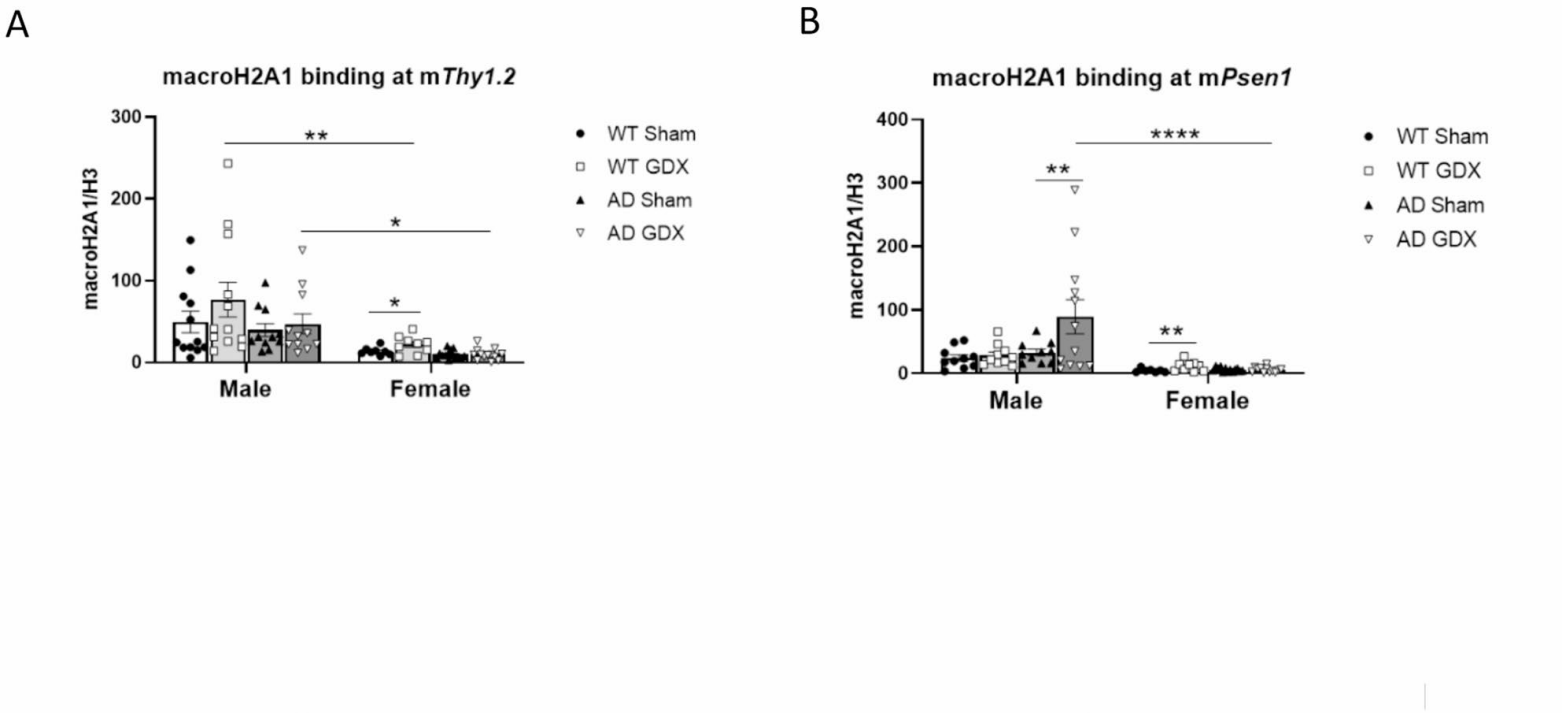
Histone variant macroH2A1 binding at the genes harbouring human mutations in male and female 3×Tg-AD mice following GDX or sham surgeries. **(A)** Binding assessment of macroH2A1 at the m*Thy1.2* promoter demonstrated that GDX increased binding in WT females only. Group sizes: males: WT Sham = 12, WT GDX = 12, AD Sham = 11, AD GDX = 11; females: WT Sham = 8, WT GDX = 9, AD Sham = 13, AD GDX = 13. **(B)** Binding assessment of macroH2A1 at the m*Psen1* promoter demonstrated that GDX increased binding in AD males and WT females. Group sizes: males: WT Sham = 10, WT GDX = 10, AD Sham = 10, AD GDX = 12; females: WT Sham = 7, WT GDX = 9, AD Sham = 13, AD GDX = 12. *q < 0.05, **q < 0.01, ***q < 0.001. Error bars represent SEM

As shown in Fig. 7A, macroH2A1 binding at the m*Thy1.2* promoter was higher in male animals than in female animals, irrespective of GDX [Sex, F(1, 46) = 23.94, *p* < 0.0001; Treatment, F(1, 46) = 1.68, *p* = 0.33; Genotype, F(1, 35) = 3.38, *p* = 0.075]. Further analysis by sex revealed that macroH2A1 binding at the promoter of the m*Thy1.2* gene was altered by GDX only in WT females, but not in their AD counterparts [Genotype: F(1, 20) = 11.02, *p* = 0.003; Treatment: F(1, 19) = 4.63, *p* = 0.04; Genotype × Treatment: F(1, 19) = 4.55, *p* = 0.046; q = 0.01 between WT Sham and WT GDX groups; q = 0.52 between AD Sham and AD GDX groups].

A three-way ANOVA for macroH2A1 binding at the promoter of the m*Psen1* gene showed main effects and interactions of sex, genotype, and treatment [Fig. 7B, Sex, F(1, 75) = 19.81, *p* < 0.0001; Genotype, F(1, 75) = 3.93, *p* = 0.05; Treatment, F(1, 75) = 4.05, *p* = 0.048; Sex × Genotype, F(1, 75) = 4.84, *p* = 0.03]. Specifically, macroH2A1 binding at the promoter of the m*Psen1* gene was higher in males than in females (q < 0.0001 between male and female AD GDX mice). GDX significantly increased macroH2A1 binding in AD males, but not in AD females (q = 0.002 between sham-treated and GDX male AD mice; q = 0.77 between sham-treated and GDX female AD mice). Nevertheless, GDX significantly increased macroH2A1 binding at m*Psen1* in female WT mice [Fig. 7B, Genotype: F(1, 37) = 1.40, *p* = 0.24, Treatment: F(1, 37) = 4.12, *p* = 0.0495, Genotype × Treatment: F(1, 37) = 5.32, *p* = 0.03. q = 0.009 between female WT Sham and female WT GDX].

#### Targeted transgene expression

**Fig. 8:**
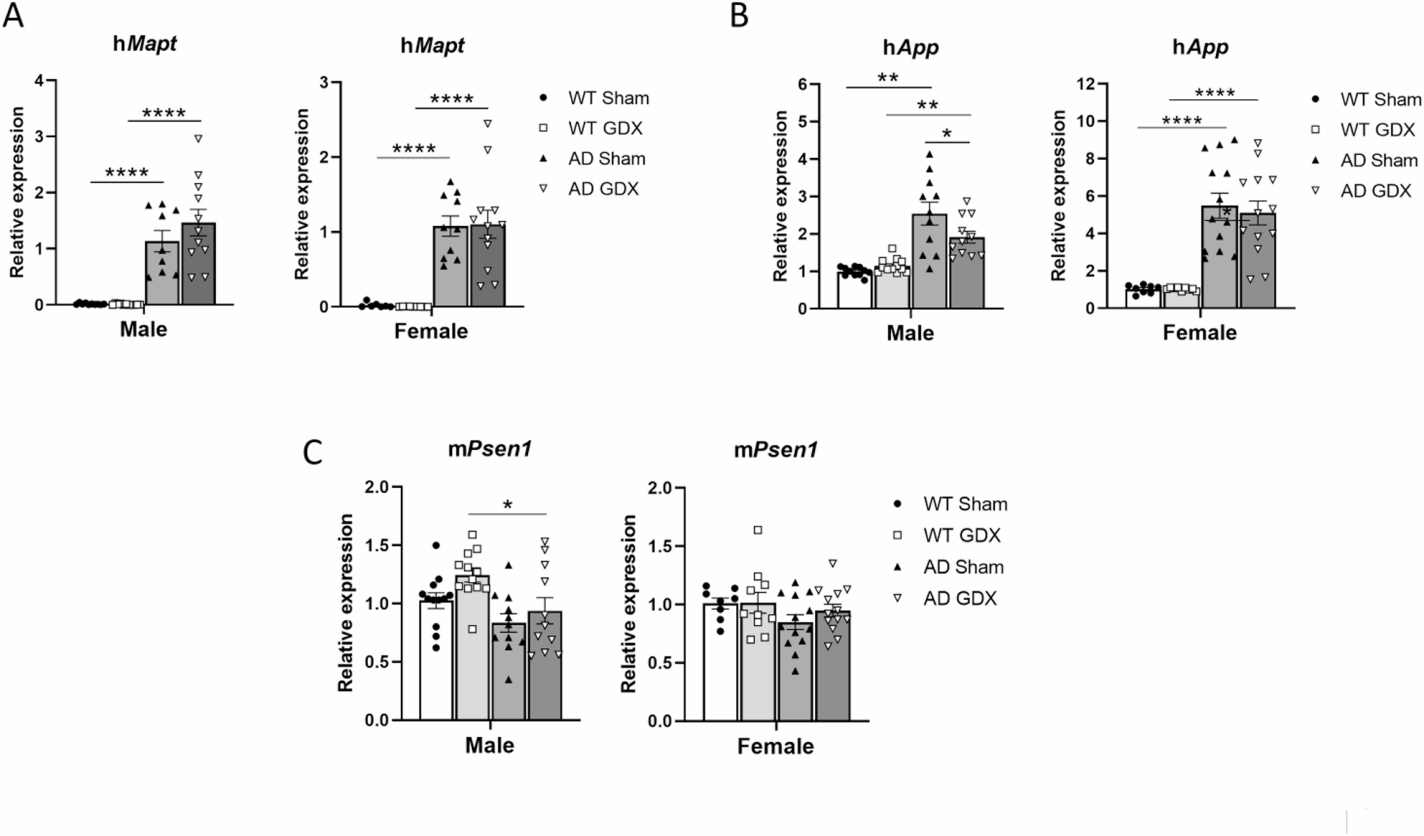
Expression of targeted mutated genes in male and female 3×Tg-AD mouse cortex by qRT-PCR following GDX or sham surgeries. **(A)** Expression of h*Mapt* carrying the human tau P301L mutation was unaffected by GDX in males (left) and females (right). Group sizes: males: WT Sham = 10, WT GDX = 11, AD Sham = 9, AD GDX = 11; females: WT Sham = 7, WT GDX = 9, AD Sham = 10, AD GDX = 12. **(B)** Expression of h*App* carrying the human Swedish AD mutation was reduced by GDX in male AD mice (left) but not female mice (right). Group sizes: males: WT Sham = 11, WT GDX = 12, AD Sham = 11, AD GDX = 11; females: WT Sham = 8, WT GDX = 9, AD Sham = 13, AD GDX = 13. **(C)** Expression of m*Psen1* carrying the human M146V mutation was unaffected by GDX in males (left) and females (right). Group sizes: males: WT Sham = 12, WT GDX = 12, AD Sham = 11, AD GDX = 11; females: WT Sham = 8, WT GDX = 10, AD Sham = 13, AD GDX = 13. Data were normalized to the geometric mean of β-actin and GAPDH. Data were then normalized to the same sex WT Sham, defined as “relative expression”.*q < 0.05, **q < 0.01, ***q < 0.001. Error bars represent SEM

Transgenic human (h)*Mapt* was specifically expressed in cortical tissue of both male and female AD mice, irrespective of GDX, and was not expressed in WT mice [Fig. 8A, Genotype: F(1, 71) = 127.5, *p* < 0.0001, Treatment: F(1, 71) = 0.61, *p* = 0.44].

As shown in Fig. 8B, GDX reduced the expression of h*App* in AD males [Genotype, F(1, 41) = 45.05, *p* < 0.0001; Treatment, F(1, 41) = 1.87, *p* = 0.18; Genotype × Treatment, F(1, 41) = 5.21, *p* = 0.03, q = 0.03] but not in AD females [Genotype: F(1, 20) = 44.53, *p* < 0.0001; Treatment, F(1, 19) = 0.15, *p* = 0.71; Genotype × Treatment, F(1, 19) = 0.15, *p* = 0.71, q > 0.999].

GDX did not affect the expression of m*Psen1* in AD mice of either sex. This gene is the endogenous mouse gene carrying a human AD mutation, under control of the endogenous mouse promoter. m*Psen1* expression was elevated only in WT GDX males when compared with AD GDX males [Fig. 8C, Genotype: F(1, 36) = 11.71, *p* = 0.006, Treatment: F(1, 46) = 4.04, *p* = 0.059, Treatment x Genotype: F(1, 20) = 0.51, *p* = 0.48, q = 0.015 between male WT GDX and male AD GDX].

## Discussion

The underlying mechanisms of the sex discrepancy in AD are not clear. Loss of steroid hormones has been suggested to contribute to AD risk in men and women (e.g., after menopause) in a sex-specific manner [90]. The lack of consensus regarding the role of steroid hormones in AD suggests that critical pathways are not yet defined and that other mechanisms, including epigenetic mechanisms which interact with gonadal hormones, are likely to underlie the influence of hormonal factors on AD.

Work on existing models rarely considers the 2:1 female/male bias in the incidence of AD, and no study to date has systematically examined the cause-effect relationship between epigenetics, behavioral dysfunction and prodromal markers of AD-like pathology in both male and female 3×Tg-AD mice, especially under the condition of surgical depletion of gonadal hormones. To test the nature of sex differences in the context of the 3×Tg-AD phenotype, the current study compared molecular and functional consequences of surgical removal of gonadal hormones in young adult male and female 3×Tg-AD and wild-type mice.

The major findings of the current study are that removal of gonadal hormones by gonadectomy (orchiectomy or ovariectomy) has significantly different consequences between male and female 3×Tg-AD animals, reflected by diverse, often opposing, changes in spatial learning and memory, neuropathology, and epigenetic modifications. Considering that adult gonadal hormones play decisive roles in specific sexual behaviors in male and female (activational effects) [28], it is likely that the major sexual hormones in male (testosterone) and female (estradiol) may influence the development of Alzheimer’s disease in different ways.

### Gonadal hormones and behavior

Our hormone assays show the efficacy of our surgical procedures. Testosterone was reduced to zero and estradiol was significantly reduced after removal of gonads. This difference in the degree of reduction between the sexes could be attributed to the different distributions of such hormones in male and female. In males, testosterone is primarily synthesized in the Leydig cells of the testes [32], whereas in premenopausal females, although estrogens are produced primarily in the ovaries, corpus luteum, and placenta, a small but significant amount of estrogens can also be produced by nongonadal organs such as the liver, heart, skin, and brain [26]. Furthermore, estrous cycles in female mice can significantly affect the levels of serum estradiol [81], and no synchronization of estrous cycle was carried out before endpoint in this study. This could result in a large variance in the levels of estradiol in the sham-operated animals. Nevertheless, we demonstrate a statistically significant reduction of estradiol levels in GDX compared to sham groups.

Our data suggest that gonadal hormones may possess some common functions beyond sex or sex-dependent roles. Some of our behavioral tests revealed sex-specific functions of gonadal hormones, while others did not. For instance, GDX significantly reduced body weight in males, but not females. In contrast, GDX increased anxiety (acrophobia in the step-down test) in both male and female 3×Tg-AD animals.

GDX also impacted sex-specific differences in spatial learning/memory in 3×Tg-AD animals, with detrimental effects particularly in females. Our MWM data show that removal of gonadal hormones from female 3×Tg-AD mice worsens spatial learning compared to males. These data are consistent with prior studies demonstrating loss of spines in ovariectomized female, but not male, rats [75]. In contrast, GDX had the opposite effect on male 3×Tg-AD animals, reflected in better performance on motor tasks (Rotarod) and spatial memory (MWM) following GDX, and implying different roles for male versus female steroid hormones in the regulation of learning and memory. Other studies have proposed that testosterone, by acting as a non-selective sigma antagonist, may produce a tonic dampening of the function of sigma receptors and consequently a decrease in NMDA receptor function that is known to play an important role in spatial learning and memory acquisition [29, 67]. This is in contrast to the well-recognized beneficial functions of estrogen, which increases the concentration of choline acetyltransferase [101], the enzyme needed for acetylcholine synthesis, and enhances communication between neurons in the hippocampus, resulting in improvements in hippocampal-mediated learning [87] and spatial memory [36] in rodents. Interestingly, one study showed that aged ovariectomized 3×Tg-AD female mice treated with leuprolide acetate, a drug used to block testosterone synthesis, exhibited significantly improved spatial learning, supporting the detrimental role of testosterone [85]. Several reports also indicated that chronic treatment with androgenic compounds impair spatial learning and memory in young and middle-aged animals [37, 43], and humans [42, 48].

Although multiple studies have shown that men and women differ on tasks of spatial cognition [58], as far as we know, our report is the first to find that GDX male and female 3×Tg-AD mice show sex-associated differences in spatial learning and memory as measured by MWM. Previous behavioral studies with GDX 3×Tg-AD mice revealed worse performance in the spontaneous alternation behavior test in both male and female animals [17, 96] and a distinct spatial memory deficit in females indicated by the MWM [32], but lack of male MWM data. In our study, the fact that only spatial learning, but not memory (as measured by the probe trial in MWM), was affected in female GDX 3×Tg-AD animals may be attributed to the independent actions of estrogen on different task systems [63].

Our study is among the first to demonstrate that the loss of gonadal hormones in male mice improves spatial learning. Furthermore, our results are consistent with human studies demonstrating that, in men, low testosterone is associated with better spatial abilities [20], and testosterone impairs long-term spatial memory [34, 43, 78]. Thus, one may speculate that while a sudden drop in estrogen, as occurs in female menopause, is detrimental to spatial learning and may contribute to the development of AD, the gradual loss of testosterone in men may be protective for spatial learning. In addition, sex-specific differences in spatial learning suggest that the increased severity of symptoms in females as compared to males with Alzheimer’s disease may be modulated by gonadal hormones.

### Sequential effect of gonadal hormones on epigenetic modifications, AD pathology and behavior

Our study documents sex-dependent differences in behavioral deficits, AD-like pathology and epigenetic changes related to AD pathology. This sex-dependent shift in phenotypic traits is not isolated to a single colony, as several independent groups have documented sex differences in 3×Tg-AD behavior [13, 14, 16, 25, 40, 88, 89, 94, 105–107], AD-like neuropathology 8, [18, 50, 86, 88, 89], response to environmental enrichment, diet, or exercise [6, 38, 41, 108], lifespan [8, 40, 92], and immunity [8, 40, 41]. Male 3×Tg-AD mice exhibit altered behavior early in life, but older female mice exhibit more severe symptoms than males. Male mice exhibit a loss or delay in AD-like pathology (https://www.jax.org/strain/004807*)* in comparison to female littermates which do not show the same loss of phenotype. Although there are no tangles at 6 months of age in either sex, there is an increase in soluble, phosphorylated tau. Western blots show increased total soluble tau and pTau-181 at 6 months of age in AD females only [60]. All these findings suggest that gonadal hormones may play distinct roles influencing AD-related pathology and behavior in a sex-dependent manner.

The nature of this sex-dependent phenomenon may be epigenetic, as we previously showed that compared to WT males, 3×Tg-AD males show increased expression of *macroH2A1* (formerly *H2afy*) [61], which codes for macroH2A1, a variant of the canonical histone H2A, important in neuroplasticity [23, 102] and often upregulated during neurodegenerative processes [33, 47, 52]). Emerging evidence suggests that histone variants are novel epigenetic regulators of memory [93], as they are critical modulators of neural plasticity [100]. As important epigenetic regulators of gene transcription, histone variant binding activities depend on their incorporation into and eviction from chromatin [97], the status of their special chaperones [71], and transcription of their coding genes. These are highly responsive to environmental stimuli, including immunosuppression [60] and age-related regulation in the brain [110]. Sex-specific effects of histone variants have also been implicated in learning and memory [93].

Following gonadectomy, the expression of the *macroH2A1* gene, coding for macroH2A1, was downregulated in male AD animals, whereas there was a trend towards upregulation in AD females. This sex-specific difference indicates that gonadal hormones regulate gene expression of *macroH2A1* in a sex-dependent manner in AD animals. However, increased mRNA expression does not necessarily translate into increased protein or increased incorporation into chromatin. Our current data revealed that decreased *macroH2A1* expression in GDX males does not alter macroH2A1 binding at the m*Mapt* or m*App* promoters.

The presence of macroH2A1 at promoter regions has been shown to inhibit gene expression [4, 15, 21, 30]. In our study, an inverse link exists between the binding activity of repressive macroH2A1 at m*Mapt* and expression of m*Mapt* in female, but not male, 3×Tg-AD mice after GDX. This suggests that female gonadal hormones may suppress the expression of the endogenous tau gene through the enhanced incorporation of macroH2A1 into chromatin at the *Mapt* gene body. Estrogen has been indicated to play protective roles against tauopathy through increasing tau dephosphorylation in human neuroblastoma cells and primary rat cortical neurons [2, 77], and in both hippocampus and cerebellum of mouse brains [91]. MacroH2A1 can regulate target gene expression in part by recruiting the transcriptional coregulator PELP1 to promote estrogen receptor-dependent transcription [54]. Estrogen is known to alleviate neurotoxicity in the brain and protect neurons [62]. So, there is reason to speculate that, in female GDX 3×Tg-AD mice, removal of estrogen may unbalance this mechanism, resulting in elevated m*Mapt* expression, contributing to the deterioration in spatial learning.

In contrast, there were no changes in macroH2A1 binding at the m*App* gene in female GDX 3×Tg-AD mice. Nevertheless, the expression of endogenous *App* was significantly elevated in female GDX 3×Tg-AD mice compared to sham-operated counterparts. Contrary to female AD mice, removal of male gonadal steroids significantly reduced the expression of endogenous *App*, again without a change in macroH2A1 binding at *App*. Our data suggest that female steroid hormones reduce endogenous *App* and tau expression (the latter by targeting macroH2A1 as a crucial regulator of the tau gene), whereas male steroid hormones play an opposing role by increasing endogenous *App*, independent of macroH2A1 regulation. On the other hand, the opposing male/female difference in *App* expression may imply that a common regulatory mechanism for App pathology, independent of macroH2A1 binding, exists in both male and female AD brain. One possibility is aromatase, which converts androgens into estrogen in the brain, independent of gonadal sources. Our data are consistent with findings of Overk et al. [84], who demonstrated an inverse relationship between aromatase and Aβ deposition in female 3xTg-AD mice. Thus, GDX in female mice may not alter brain Aβ levels in our study due to the presence of aromatase [84]. Other studies indicate the importance of female gonadal hormones in limiting the spread of neuropathology in AD, as GDX reduces the activity of α-secretase and neprilysin and increases the activity of β-secretase [45]. Although estrogen reportedly upregulates expression of γ-secretase in cultured human neuronal and glial cells [82], no significant effect of GDX on γ-secretase protein expression was observed in sheep brain [9], consistent with our observations of m*Psen1* expression in the 3×Tg-AD mouse model.

A previous study has indicated that in 3×Tg-AD animals, murine tau is hyperphosphorylated and promotes neurofibrillary tangle formation by co-aggregating with transgenic tau [7]. An in vitro study also revealed that wild type tau could alter the transcription and alternative polyadenylation profiles of numerous nuclear precursor mRNAs, which then translate to form proteins involved in chromatin remodeling and splicing [76]. Therefore, upregulated m*Mapt* gene expression, possibly followed by a high level of endogenous tau protein expression, may contribute to maintaining a high level of tau pathology after removal of female steroid hormones. On the other hand, the upregulated m*App* following GDX in females may also aggravate amyloid pathology, as a study comparing APP/PS1 mice with and without the endogenous mouse *App* gene indicated that the additional mouse Aβ increased soluble amyloid accumulation [57].

In contrast to the endogenous AD-related genes, binding of macroH2A1 to the Thy1 expression cassette that is upstream of human *Mapt* and *App* genes did not result in any significant differences in expression of these two genes in GDX female AD animals compared to sham-treated AD females. Binding of macroH2A1 to the Thy1 promoter was greater in males than in females, however, which may account for the reduced expression of the h*App* transgene in AD males compared to females. In contrast, GDX male AD mice did exhibit (1) reduced expression of the *App* transgene compared to sham-operated mice without altered macroH2A1 binding at the Thy1 gene and (2) a significant increase of macroH2A1 binding at the *Psen1* gene without a change in *Psen1* gene expression. The fact that reduced *App* transgene expression occurred in male AD GDX mice in the absence of a difference in macroH2A1 binding at the Thy1 expression cassette, and that the increased macroH2A1 binding to *Psen1* did not alter *Psen1* gene expression in male AD GDX mice compared to sham-operated animals, implies the existence of additional regulatory factors other than macroH2A1.

Taken together, our data further confirm the differential effects of male and female gonadal steroids on AD-related pathology: male hormones mainly target endogenous and transgenic *App* independent of macroH2A1 regulation, whereas female hormones target both endogenous macroH2A1-dependent m*Mapt* regulation and macroH2A1-independent m*App* regulation, but do not influence transgene expression. Supporting our observations, one study observed that after GDX of aged mice with elevated levels of *App* mRNA, *App* was downregulated in males, but upregulated in females [103]. In the same study, supplementation with estradiol decreased App levels in ovariectomized females, whereas testosterone supplementation increased App mRNA levels in castrated males. Thus, gonadal steroids influence sex-dependent differences in AD-like pathology in 3×Tg-AD mice.

GDX in mouse models of AD may approximate a critical aspect of human reproductive aging in males and females by depletion of gonadal hormones in the adult. In females, acute, surgical depletion of gonadal hormones approximates the sudden drop accompanying menopause. However, in males, acute GDX poorly mimics the timing of the slow and progressive reduction in male hormones with age. Whether the rate of hormone decline influences AD-related outcomes is an unknown factor. Compensatory neuroendocrine responses of increased luteinizing hormone (LH) and follicle stimulating hormone (FSH) following removal of gonads should be also considered [31]. These compensatory changes occur in both simulated and true reproductive aging, and therefore in this aspect, GDX in mouse models remains relevant to the human condition [31]. However, in GDX rodents, the levels of serum FSH and LH are rapidly and significantly elevated post surgery [95], mimicking the rapid menopausal changes in women better than the more gradual changes in aging men [51].

Here, depletion of hormones in young adults (at 3 months of age in this study) rather than in aged animals is an accurate model for oophorectomy for ovarian cancer or endometriosis and for prostatectomy for prostate cancer. A series of studies during the last 2 decades have shown unequivocally that bilateral oophorectomy at 45 years of age or earlier is associated with higher risk of cognitive impairment and dementia [39]. Unlike oophorectomy however, it remains unclear whether androgen deprivation therapy is associated with subsequent dementia risk in patients with prostate cancer [53]. Two studies reported no relationship of anti-androgens to Alzheimer’s disease or cognitive disorder [66], whereas another study showed that chemical castration doubles the risk of Alzheimer’s disease in patients with prostate cancer [80]. A recent study utilizing a large cohort of patients with prostate cancer proposed that antiandrogen monotherapy, but not orchiectomy or gonadotropin-releasing hormone agonists, was associated with increased dementia risk, implying different effects of surgical vs. chemical castration [53].

## Conclusions

Three-month-old 3×Tg-AD mice were gonadectomised to explore the role of steroid hormones in sex-dependent discrepancies in AD and the interaction of gonadal hormones with behavior, neuropathological AD-like protein markers and transcriptional and functional changes in epigenetic factors. We show that removal of gonadal hormones results in sex-dependent differences in all these aspects. Gonadectomised AD females performed more poorly in a spatial learning task, which was associated with elevated endogenous m*Mapt* and m*App* expression. In contrast, gonadectomized AD males exhibit improved spatial learning and memory along with reduced endogenous and transgenic *App* expression. Consistent with the behavioral changes, gonadectomised AD females displayed worse AD-related pathology than gonadectomised AD males. Such sex-related differences led us to further explore the sex-dependent regulation of AD-related pathology. The mRNA expression of the *macroH2A1* gene was downregulated in male AD mice after GDX, and the binding of macroH2A1 to the m*Mapt* gene was downregulated in female mice after GDX, suggesting changes in incorporation of macroH2A1 into nucleosomes. GDX enhanced expression of m*Mapt* and m*App* genes in AD females, consistent with reduced binding activity of the repressive histone variant macroH2A1 at the m*Mapt* gene body, compared to their sham counterparts. Increased expression of genes coding for amyloid precursor protein and tau was consistent with worse learning and memory deficits following removal of female gonadal steroids. In contrast, gonadectomized AD males showed significantly reduced expression of the *App* gene, reduced cortical soluble Aβ42 levels, and increased macroH2A1 binding at the m*Psen1* promoter (without leading to altered expression of m*Psen1* mRNA), compared to sham-operated AD males. All these GDX-induced changes support that, in 3xTg-AD mice, male gonadal hormones act on spatial learning and memory functions through different mechanisms than females. In sum, female gonadal hormones enhance spatial learning, whereas male gonadal hormones are detrimental to spatial learning and memory. Furthermore, female gonadal hormones may be beneficial by decreasing expression of the endogenous m*Mapt* and m*App* genes. Decreased levels of female steroids after GDX may contribute to the increased plaque and tangle load in females, but not males. Conversely, male gonadal hormones may increase *App* expression and Aβ_42_ levels but have no effect on tau expression. Our work suggests that adult gonadal hormones contribute to sex differences in AD pathology and learning and memory in AD, and that sex-specific differences in AD pathology are at least partially due to the action of histone variants such as macroH2A1.

## Data Availability

The datasets used and/or analysed during the current study are available from the corresponding author on reasonable request.

## Abbreviations

3×Tg-AD: Triple transgenic mouse model of Alzheimer’s disease
AD: Alzheimer’s disease
Aβ: Amyloid-beta
ANOVA: Analysis of variance
ELISA: Enzyme-linked immunosorbent assay
GDX: Gonadectomy
FSH: Follicle stimulating hormone
IPC: Internal positive control
LH: Luteinizing hormone
mH2A1: MacroH2A1
MWM: Morris water maze
PBS: Phosphate-buffered saline
pTau 181: Phospho- tau 181
pTau: Phosphorylated tau
qRT-PCR: Quantitative reverse transcription polymerase chain reaction
tTau: Total tau
TBS: Tris-buffered saline
WT: Wild-type
